# Assessing the Effects of Symmetry on Motif Discovery and Modeling

**DOI:** 10.1371/journal.pone.0024908

**Published:** 2011-09-20

**Authors:** Lala M. Motlhabi, Gary D. Stormo

**Affiliations:** Department of Genetics, Washington University School of Medicine, St. Louis, Missouri, United States of America; Semmelweis University, Hungary

## Abstract

**Background:**

Identifying the DNA binding sites for transcription factors is a key task in modeling the gene regulatory network of a cell. Predicting DNA binding sites computationally suffers from high false positives and false negatives due to various contributing factors, including the inaccurate models for transcription factor specificity. One source of inaccuracy in the specificity models is the assumption of asymmetry for symmetric models.

**Methodology/Principal Findings:**

Using simulation studies, so that the correct binding site model is known and various parameters of the process can be systematically controlled, we test different motif finding algorithms on both symmetric and asymmetric binding site data. We show that if the true binding site is asymmetric the results are unambiguous and the asymmetric model is clearly superior to the symmetric model. But if the true binding specificity is symmetric commonly used methods can infer, incorrectly, that the motif is asymmetric. The resulting inaccurate motifs lead to lower sensitivity and specificity than would the correct, symmetric models. We also show how the correct model can be obtained by the use of appropriate measures of statistical significance.

**Conclusions/Significance:**

This study demonstrates that the most commonly used motif-finding approaches usually model symmetric motifs incorrectly, which leads to higher than necessary false prediction errors. It also demonstrates how alternative motif-finding methods can correct the problem, providing more accurate motif models and reducing the errors. Furthermore, it provides criteria for determining whether a symmetric or asymmetric model is the most appropriate for any experimental dataset.

## Introduction

Transcription is a key step in gene expression and its regulation. The transcription initiation reaction is facilitated by *cis*-regulatory regions containing DNA sequence motifs which are binding sites for general and/or specific transcription factors [1], [2], [3]. In order for the right gene to be expressed at the right place and time and at the right level, a high degree of specificity during protein-DNA recognition events is required to recruit the transcriptional machinery. The challenging task of identifying *cis*-regulatory elements often suffers from high false positive and false negative rates. One contributing factor to the error rate is inaccurate models of transcription factor specificity. The convergence of *in vivo* experimental approaches and computational methods can help in identifying motifs for a particular transcription factor [4], but critical issues related to motif discovery approaches need to be addressed.

Large genomic scale experimental approaches that determine the genomic locations of binding sites for specific transcription factors, such as ChIP-chip and ChIP-Seq assays [5], [6], [7], [8], [9], are sufficient for many overall characteristics of regulatory networks, such as the connectivity between regulatory factors and the genes they regulate. But having a model for the specificity of the transcription factor allows one to have a finer scale resolution of the binding sites [4], [10], [11], [12] and to infer the effects of genetic variations on gene expression [13], [14]. Most specificity models employ position weight matrices (PWMs) [15], [16], [17] although more complex models can be used if needed [18]. A variety of motif discovery algorithms have been developed to predict the binding site specificity of a transcription factor based on collections of sequences containing binding sites (reviewed in [4], [16], [19], [20], [21]).

**Figure 1:**
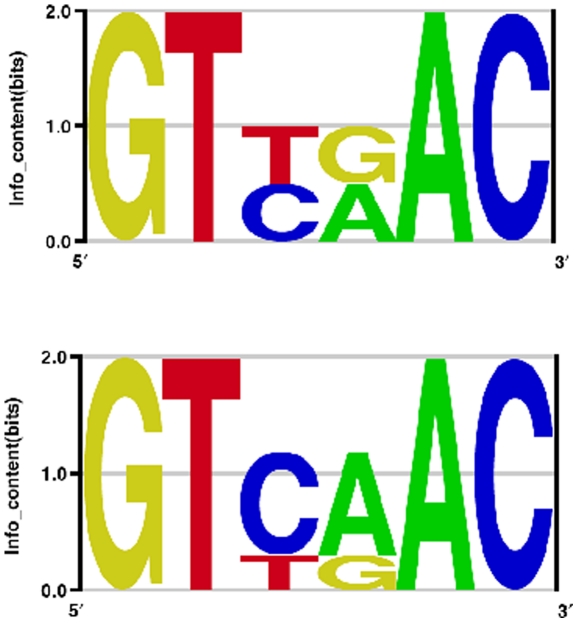
Logos for HincII restriction enzyme. Top, the Logo for the true specificity of the HincII restriction enzyme. Bottom, the Logo for an incorrect motif in which one of the asymmetric sites (GTTGAC) has been selected in the opposite orientation (GTCAAC) to create an asymmetric motif.

Since most transcription factors can affect gene regulation in either orientation, motif discovery algorithms generally search both strands of the DNA regions to find the common motif. But there are a large number of transcription factors that bind DNA as homo-dimers, in which case the binding site is often symmetric, or at least approximately symmetric. A symmetric motif does not imply that each individual binding site is symmetric, merely that the consensus sequence is and that changes in affinity due to variations from the consensus should be equivalent in both halves of the site. Motif discovery algorithms that search both strands for binding sites, but don't require symmetry, will often find incorrect, approximately symmetric motifs. This is easily demonstrated using the *HincII* restriction enzyme (Figure 1) as an example. Its recognition site is GTYRAC (Y = C/T, R = A/G) which matches four distinct DNA sites, two of them perfectly symmetric (GTTAAC and GTCGAC) and two of them asymmetric (GTCAAC and GTTGAC). A motif discovery algorithm that allows either orientation of the sites will use the opposite orientation of one of the asymmetric sites to generate a motif that is asymmetric (Figure 1 bottom). This is clearly an inaccurate model for the motif, although for a restriction enzyme where the activity is all-or-none for sites that either match or not, it would not affect the prediction of sites. But for transcription factors, where variations in binding affinity can be important for proper regulation, such an inaccurate model could lead to loss of sensitivity and specificity in binding site predictions. The issue of symmetric binding sites has been addressed many times before, and most motif finding algorithms allow the user to constrain the search for symmetric patterns (e.g. [22], [23], [24], [25], [26], [27]). However, it is usually left to the user of those programs to determine the motif they find most convincing and any artifacts that they report are often propagated to motif databases. To highlight this issue and propose a solution we use simulation studies to demonstrate the problems associated with motif discovery on symmetric sites and how to select the most accurate model.

## Methods

### Binding Site Models

**Figure 2:**
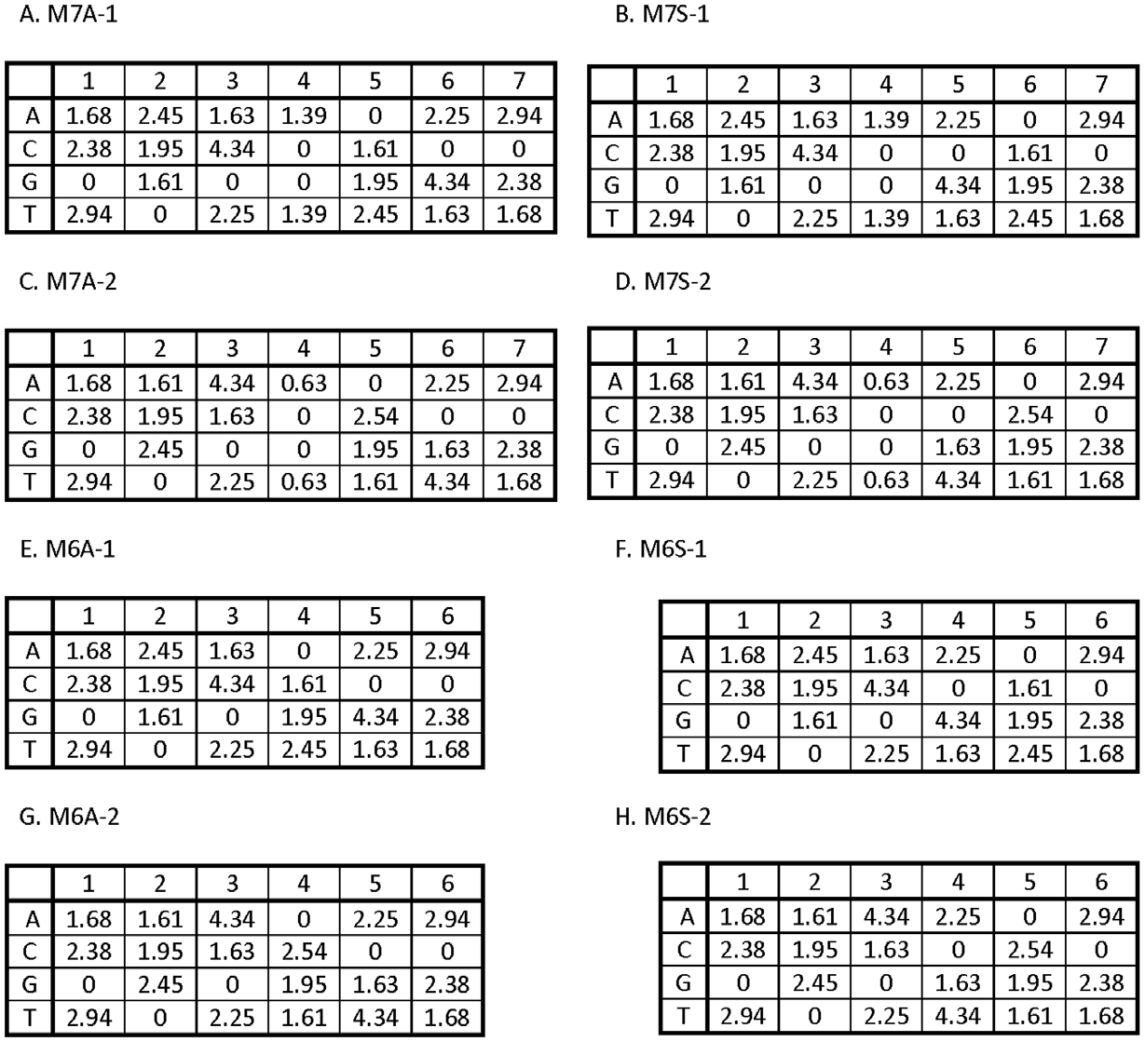
The energy matrices for true binding site models. Each position has a single base with 0 energy which is the preferred base, and all of the other bases increase the binding energy by the amount shown. The top four matrices are for 7-long binding sites and the bottom four are for 6-long matrices. The left column are all asymmetric matrices and the right column are all symmetric. The parameters in each pair (row) of matrices are the same, but two of the position (column) orders are changed between the left and right matrix.

Binding site models are derived from the experimentally measured and characterized Mnt protein of salmonella phage P22 [28]. Mnt is a repressor that binds as a tetramer, with each dimer binding to a nearly symmetric seven base-pair half-site with a consensus of GTGGACC. If positions five and six are switched it becomes the symmetric site GTGGCAC, (this is an “odd symmetry” with a central base not included in the symmetry; the other strand is GTGCCAC, so the symmetric consensus is GTGSCAC, where S = G/C). To compare the performance of different algorithms we have created eight different variants of the Mnt motif that are used as “true motifs” from which sample binding sites are obtained for motif discovery (Figure 2). Four of the true motifs are seven-long, having either the Mnt-like asymmetric consensus of GTGSACC (M7A-1 and M7A-2) or the symmetrized version GTGSCAC (M7S-1 and M7S-2) in which the fifth and sixth motif positions are exchanged but all of the parameters remain the same. In the other four of the true motifs the central base is deleted to create two asymmetric 6-long motifs with a consensus of GTGACC (M6A-1 and M6A-2) and two with an “even symmetry”, a completely symmetric model with a consensus of GTGCAC (M6S-1 and M6S-2). The differences between the two models of each type (“-1” vs “-2”) are variations in the degree of symmetry. For example, position 2 of M7A-1 has the affinity ranks of T,G>C,A, whereas M7A-2 has affinity ranks T,A>C,G. The set of energies in each position are the same except for the center position of the 7-long matrices where there is less specificity (differences in affinity) between the bases in “-2” models. These differences affect the propensity for choosing the orientation of sites in asymmetric models (see RESULTS).

### DNA binding site sampling

For each of the energy matrices of Figure 2 we generated random samples of 500 binding sites. The probability of any specific 6- or 7-long sequence, S_i_, depends on its binding energy, E_i_, as specified by energy matrix, using the standard biophysical model for binding [29], [30], [31]:
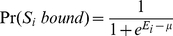
(1)where μ is the chemical potential of the DNA-binding protein (related to its concentration). For our simulations we define the binding energy of the consensus sequence as 0 (Figure 2) and set the μ value to −0.5 such that the consensus sequence has binding probability of 0.38. This means that the ratio of every other sequence to the consensus will be very nearly equal to the ratios of their binding affinities. The sets of all the sampled binding sites and their energies are provided in Table S1.

### Motif finding and significance testing

Each set of sequences (Table S1) was analyzed by the motif discovery program Consensus [32], [33]. In this case the motif discovery problem is trivial and any other program that finds a model which maximizes the probability of the data, such as by Expectation Maximization (EM) or Gibbs' sampling [25], [34], would return nearly identical results. Using Consensus it is easy to test three different modes of motif finding. In the first mode (runtime parameter -c0) the sites are just taken as given. This serves as a control because the discrepancy between its discovered motif and the true motif is due only to the limited sample size (500 sites) and the difference in binding probability between the assumed probabilistic model of the algorithm and the biophysical one for the site sampling [29], [30] which is quite small at the value of μ used. The second mode of motif finding (runtime parameter -c2) allows every individual site to be selected in either of its two possible orientations. If the true motif is asymmetric this mode will rarely choose the wrong orientation so the result should be nearly identical with mode -c0. But if the site is symmetric it has the risk of creating an incorrect motif as shown for *HincII* sites in the Introduction (Figure 1). The third mode allowed by Consensus (runtime parameter -c3) assumes that the binding motif is symmetric and therefore every site is really two sites, one in each orientation, which are combined to derive the motif model. In this case the sample size is doubled to 1000 sites and the complementary parameters in the symmetric positions of the model are constrained to be identical.

### Assessment of motif accuracy

Since we know the correct motif for each of the samples, we can assess which method of predicting the motif, by assuming asymmetry or assuming symmetry, works best for each sample. We can compare the resulting motifs visually by creating Logos from the aligned binding sites [35], [36]. We can also measure the information content of the aligned binding sites [16], [37]. Information content, or a very similar measure, is used in many motif discovery algorithms, such as Consensus, EM, and Gibbs' sampler [16], [25], [33], [34] as the criterion to select the most significant alignment. We can also determine an E-value for each of the discovered motifs, which is the number of motifs expected by chance with an information as high, or higher, than that found given the number of sequences and the number of possible alignments (and taking the background base probabilities into account, which in this case are set to 0.25 for each base). The E-value reported by the Consensus program is based on the combination of two types of information. One is the p-value of obtaining a PWM with the information content equal to, or higher than, that observed from a random alignment of sequences with the background composition, determined from an extreme value distribution analysis [32], [38], [39]. That p-value for the PWM is then converted to a E-value by taking into account the number of possible alignments of the of the input dataset [32]. In every case the motifs are extremely significant and we report the −ln(E-value) so that larger values are more significant.

Finally, since we know the true motif we can calculate the true binding energy for all possible sequences (there are 4096 6-long sequences and 16,384 7-long sequences) and compare those to the predicted binding energies from each of the discovered motifs. For the probability of the factor binding to a site S_i_ we used the sum of it binding in either orientation, then we compared, using R^2^ (the square of the Pearson correlation coefficient), the logarithm of that sum for the true binding energies and the predicted binding energies for each model. If instead of using the sum we used the maximum of the two orientations, the R^2^ values in general were decreased by 0.01 to 0.1 (data not shown).

## Results

**Figure 3:**
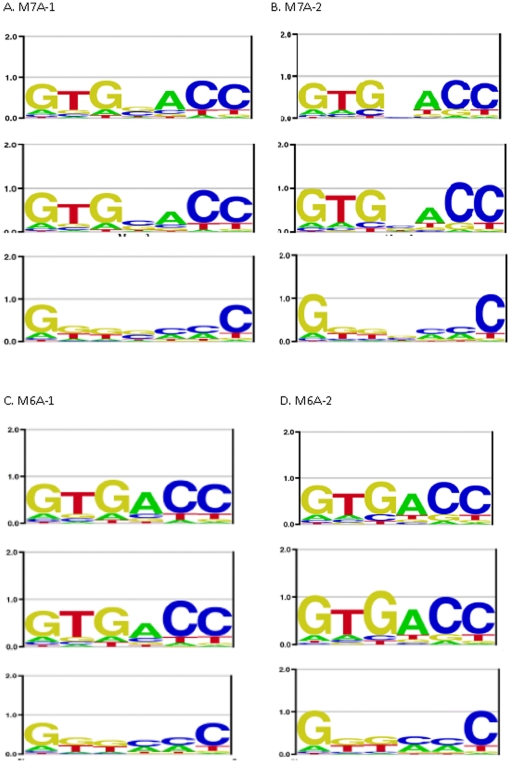
The Logos for each of the asymmetric motifs. True asymmetric motifs (top one in each set) and the Logos for the motifs discovered using either the asymmetric model (middle one in each set) or the symmetric model (bottom one in each set).

**Figure 4:**
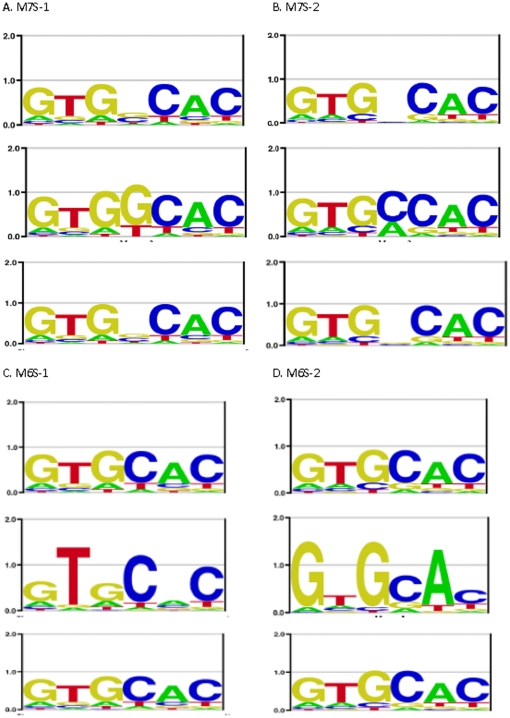
The Logos for each of the symmetric motifs. True symmetric motifs (top one in each set) and the Logos for the motifs discovered using either the asymmetric model (middle one in each set) or the symmetric model (bottom one in each set).

Figures 3 and 4 compares the logos for the true motifs and the motifs generated by the asymmetric model and symmetric model, respectively, for each data set (the motif generated by the correct alignment of sites is nearly identical to the true motif in every case and is not shown). Table 1 provides the information content for each motif as well as the −ln(E-value). It can be seen that if the true motif is asymmetric the motif obtained from the asymmetric mode of the program is very accurate; sometimes it has slightly more information content than the true model just because the true motif is approximately symmetric and occasionally a site will score slightly higher in the reverse orientation from how it was generated. The symmetric models, when the true motif is asymmetric, are quite poor and have much lower information content and −ln(E-value) than the asymmetric models for the same datasets.

**Table 1:** Information content and −ln(E-value) for predicted matrices.

	M7A-1	M7A-2	M6A-1	M6A-2	M7S-1	M7S-2	M6S-1	M6S-2
**Info Content**								
True	3.2	3.1	3.1	3.1	3.2	3.1	3.1	3.1
Asym	**3.3**	**3.1**	**3.2**	**3.1**	3.9	3.8	3.4	3.4
Sym	2.1	1.7	2.1	1.7	**3.2**	**3.1**	**3.1**	**3.1**
**−ln(E-value)**								
Asym	**1227**	**1163**	**1189**	**1161**	1536	1510	1326	1313
Sym	1015	830	1018	849	**1560**	**1534**	**1520**	**1510**

When the true model is symmetric the results are quite different and highlight the problem of analyzing symmetric sites under the assumption of asymmetry. The logos clearly show that the symmetric models are quite accurate whereas the asymmetric ones are not. But the information content of the asymmetric model is higher, similar to the *HincII* example of Figure 1 but now shown for a realistic binding site model with variable affinities for different sequences. Since one applies motif finding algorithms to datasets with unknown motifs one cannot evaluate which is correct simply by comparing the logos, and in this case the information content gives a misleading conclusion. Since most motif discovery programs define the most significant motif as the one with the highest information content, or some related likelihood ratio statistic, they would get the wrong answer on all of these symmetric motifs. However, by comparing E-values one can obtain the correct answer. The E-value depends on both the significance of the alignment, as measured by the information content of the sites, as well as the number of possible alignments. In the case of the symmetric model each site has only one alignment (because both orientations are used simultaneously for that alignment), whereas the asymmetric model allows each site to occur in either of two orientations, therefore there are 2^N^ possible choices for N sequences. By correcting for that much larger set of possible alignments, the E-value ranking is a more accurate measure of the statistical significance and can obtain the correct model even in cases where it has lower information content.

Given a matrix for a transcription factor one can predict binding sites in a genome by scoring each possible site. One may use a threshold and predict as binding sites those whose score exceeds the cutoff, or one can use a quantitative prediction of the probability of binding based on the score. Quantitative scores are especially useful when one expects there are multiple binding sites close together because one can sum the predicted probabilities to get an “occupancy” score for the region being considered [40]. In either case, the accuracy of the predicted motif will affect the false positive and false negative predictions of regulatory regions. To determine the accuracy of each model we calculated the binding energy for all possible binding sites based on the true energy model and compared those to the binding energies predicted by each model. We use R^2^, the square of the Pearson correlation coefficient which indicates what fraction of the true variance in binding energy is captured by the model, as the measure of accuracy. Table 2 shows the R^2^ values for each predicted matrix for each dataset. The control matrix, in which the correct orientations of each binding site are known, indicates the best expected accuracy given the sample size of 500 sites and the fact that the log-odds probability model does not match the biophysical model exactly. In general these R^2^ values are quite high, all but one being over 0.93 and those for the symmetric sites being between 0.96 and 0.99. When the sites are asymmetric the asymmetric model does essentially as well as could be expected (values in bold), but the symmetric model is quite poor. When the true motif is symmetric, the predicted model based on the assumption of symmetry is very accurate (values in bold), sometimes even better than the control model because the sample size is twice as large (each site contributes to the model in both orientations). The model based on the asymmetric assumption is highly variable; with the 7-long motifs in these examples it is not much worse than the symmetric model but for the 6-long motifs it is significantly worse. These results are consistent with the E-value analysis presented above and show that the assumption of asymmetry when sites are truly symmetric can be misleading and decrease the accuracy of binding site predictions considerably.

**Table 2:** R^2^ between predicted energies and true energies.

	M7A-1	M7A-2	M6A-1	M6A-2	M7S-1	M7S-2	M6S-1	M6S-2
Control	0.93	0.88	0.94	0.94	0.98	0.98	0.99	0.96
Asym	**0.92**	**0.89**	**0.94**	**0.93**	0.97	0.97	0.80	0.80
Sym	0.36	0.31	0.34	0.33	**0.99**	**0.98**	**0.99**	**0.97**

## Discussion

There are now many different approaches to study DNA-protein interactions and the specificity of transcription factors, both using *in vivo* location analysis (such as ChIP-chip and ChIP-Seq) and several different types of high-throughput *in vitro* binding assays [7], [8], [9], [10], [11], [41]. Most of those data sources do not identify the binding sites or recognition motifs directly, but rely on some type of motif discovery program to determine the specificity of the transcription factor. In several recent studies we demonstrated that the accuracy of the discovered motif can vary considerably depending on the type of discovery algorithm used, and that different data types may require specialized analysis methods to maximize the accuracy [27], [30], [31], [42]. But an over-riding issue that affects every motif discovery method, even those that use more complex models than PWMs, is whether the specificity is symmetric. Many transcription factors bind as homo-dimers and in such cases one expects the binding sites may be symmetric. But if the program employed does not specifically assume symmetry it can (nearly) always find an alignment of the sites that is nearly symmetric but has slightly more information content than the completely symmetric motif. It is quite common in publications to see Logos of motifs that appear approximately symmetric, and even for the text to say something like ‘the discovered motif is nearly symmetric but the left half is somewhat more conserved than the right half’. We suspect that most, or all, of those cases are artifacts of the motif discovery algorithm and that the true motif is likely to be symmetric. We encourage the database curators to take this issue seriously and to assess whether the asymmetric model is more significant than the symmetric one, which requires more than just a comparison of their information contents or similar likelihood ratio statistics. The users of transcription factor motif databases can perform such assessments themselves if the raw data are made available. We presented an E-value based method that takes into account the number of possible alignments as one way to estimate the relative statistical significance of the two models. An easier approach that can also work is to simply take into account that the symmetric model has only half as many free parameters as the asymmetric one (for the same length motif) because of the constraints imposed by the symmetry, and to estimate the statistical significance taking into account the number of parameters being fit.

The accuracy of the motif for transcription factor specificities is not a trivial problem. Even small differences in the models can lead to large differences in the sensitivities and specificities, the false positive and negative rates, when predicting sites in a genome [29], [30]. Therefore we recommend that motif discovery algorithms be applied in both asymmetric and symmetric discovery modes and that the conclusions be based on sound statistical evaluations of their relative significance.

## Supporting Information

Table S1 **Selected sites and their energies from each of the eight binding site models.**
(TXT)

## References

[1] Ptashne M, Gann A (1997). Transcriptional activation by recruitment.. Nature.

[2] Smale ST, Kadonaga JT (2003). The RNA polymerase II core promoter.. Annu Rev Biochem.

[3] Orphanides G, Reinberg D (2002). A unified theory of gene expression.. Cell.

[4] Elnitski L, Jin VX, Farnham PJ, Jones SJ (2006). Locating mammalian transcription factor binding sites: a survey of computational and experimental techniques.. Genome Res.

[5] Dorschner MO, Hawrylycz M, Humbert R, Wallace JC, Shafer A (2004). High-throughput localization of functional elements by quantitative chromatin profiling.. Nat Methods.

[6] ENCODE (2004). The ENCODE (ENCyclopedia Of DNA Elements) Project.. Science.

[7] Johnson DS, Mortazavi A, Myers RM, Wold B (2007). Genome-wide mapping of in vivo protein-DNA interactions.. Science.

[8] Kharchenko PV, Tolstorukov MY, Park PJ (2008). Design and analysis of ChIP-seq experiments for DNA-binding proteins.. Nat Biotechnol.

[9] Park PJ (2009). ChIP-seq: advantages and challenges of a maturing technology.. Nat Rev Genet.

[10] Ji H, Jiang H, Ma W, Johnson DS, Myers RM (2008). An integrated software system for analyzing ChIP-chip and ChIP-seq data.. Nat Biotechnol.

[11] Pepke S, Wold B, Mortazavi A (2009). Computation for ChIP-seq and RNA-seq studies.. Nat Methods.

[12] Taslim C, Wu J, Yan P, Singer G, Parvin J (2009). Comparative study on ChIP-seq data: normalization and binding pattern characterization.. Bioinformatics.

[13] Maston GA, Evans SK, Green MR (2006). Transcriptional regulatory elements in the human genome.. Annu Rev Genomics Hum Genet.

[14] Wray GA (2007). The evolutionary significance of cis-regulatory mutations.. Nat Rev Genet.

[15] Sandelin A, Alkema W, Engstrom P, Wasserman WW, Lenhard B (2004). JASPAR: an open-access database for eukaryotic transcription factor binding profiles.. Nucleic Acids Res.

[16] Stormo GD (2000). DNA binding sites: representation and discovery.. Bioinformatics.

[17] Matys V, Kel-Margoulis OV, Fricke E, Liebich I, Land S (2006). TRANSFAC and its module TRANSCompel: transcriptional gene regulation in eukaryotes.. Nucleic Acids Res.

[18] Stormo GD (2011). Maximally efficient modeling of DNA sequence motifs at all levels of complexity.. Genetics.

[19] Das MK, Dai HK (2007). A survey of DNA motif finding algorithms.. BMC Bioinformatics.

[20] D'Haeseleer P (2006). How does DNA sequence motif discovery work?. Nat Biotechnol.

[21] GuhaThakurta D (2006). Computational identification of transcriptional regulatory elements in DNA sequence.. Nucleic Acids Res.

[22] Bailey TL, Elkan C (1995). The value of prior knowledge in discovering motifs with MEME.. Proc Int Conf Intell Syst Mol Biol.

[23] Hertz GZ, Hartzell GW, Stormo GD (1990). Identification of consensus patterns in unaligned DNA sequences known to be functionally related.. Comput Appl Biosci.

[24] Kechris KJ, van Zwet E, Bickel PJ, Eisen MB (2004). Detecting DNA regulatory motifs by incorporating positional trends in information content.. Genome Biol.

[25] Lawrence CE, Reilly AA (1990). An expectation maximization (EM) algorithm for the identification and characterization of common sites in unaligned biopolymer sequences.. Proteins.

[26] Liu X, Brutlag DL, Liu JS (2001). BioProspector: discovering conserved DNA motifs in upstream regulatory regions of co-expressed genes.. Pac Symp Biocomput.

[27] Zhao Y, Stormo GD (2011). Quantitative analysis demonstrates most transcription factors require only simple models of specificity.. Nat Biotechnol.

[28] Fields DS, He Y, Al-Uzri AY, Stormo GD (1997). Quantitative specificity of the Mnt repressor.. J Mol Biol.

[29] Djordjevic M, Sengupta AM, Shraiman BI (2003). A biophysical approach to transcription factor binding site discovery.. Genome Res.

[30] Homsi DS, Gupta V, Stormo GD (2009). Modeling the quantitative specificity of DNA-binding proteins from example binding sites.. PLoS One.

[31] Zhao Y, Granas D, Stormo GD (2009). Inferring binding energies from selected binding sites.. PLoS Comput Biol.

[32] Hertz GZ, Stormo GD (1999). Identifying DNA and protein patterns with statistically significant alignments of multiple sequences.. Bioinformatics.

[33] Stormo GD, Hartzell GW (1989). Identifying protein-binding sites from unaligned DNA fragments.. Proc Natl Acad Sci U S A.

[34] Lawrence CE, Altschul SF, Boguski MS, Liu JS, Neuwald AF (1993). Detecting subtle sequence signals: a Gibbs sampling strategy for multiple alignment.. Science.

[35] Schneider TD, Stephens RM (1990). Sequence logos: a new way to display consensus sequences.. Nucleic Acids Res.

[36] Workman CT, Yin Y, Corcoran DL, Ideker T, Stormo GD (2005). enoLOGOS: a versatile web tool for energy normalized sequence logos.. Nucleic Acids Res.

[37] Schneider TD, Stormo GD, Gold L, Ehrenfeucht A (1986). Information content of binding sites on nucleotide sequences.. J Mol Biol.

[38] Nagarajan N, Jones N, Keich U (2005). Computing the P-value of the information content from an alignment of multiple sequences.. Bioinformatics.

[39] Nagarajan N, Keich U (2008). FAST: Fourier transform based algorithms for significance testing of ungapped multiple alignments.. Bioinformatics.

[40] Granek JA, Clarke ND (2005). Explicit equilibrium modeling of transcription-factor binding and gene regulation.. Genome Biol.

[41] Stormo GD, Zhao Y (2010). Determining the specificity of protein-DNA interactions.. Nat Rev Genet.

[42] Christensen RG, Gupta A, Zuo Z, Schriefer LA, Wolfe SA (2011). A modified bacterial one-hybrid system yields improved quantitative models of transcription factor specificity.. Nucleic Acids Res.

